# Supplementary material for: Strongly Improving the Sensitivity of Phosphorescence-Based Optical Oxygen Sensors by Exploiting Nano-Porous Substrates
Source: Biosensors (Basel). 2022 Sep 20;12(10):774. doi: 10.3390/bios12100774 (PMC9599114; doi:10.3390/bios12100774)
Supplement: Supplementary file 1 [file biosensors-12-00774-s001.zip › biosensors-1885469-supplementary.pdf]

Supporting Information

Strongly improving the sensitivity of phosphorescence-based optical oxygen sensors by exploiting nano-porous substrates

*Chih-Yi Liu, Annada Sankar Sadhu, Riya Karmakar, Cheng-Shane Chu, Yi-Nan Lin, Shih-Hsin Chang, Goutam Kumar Dalapati, Sajal Biring**

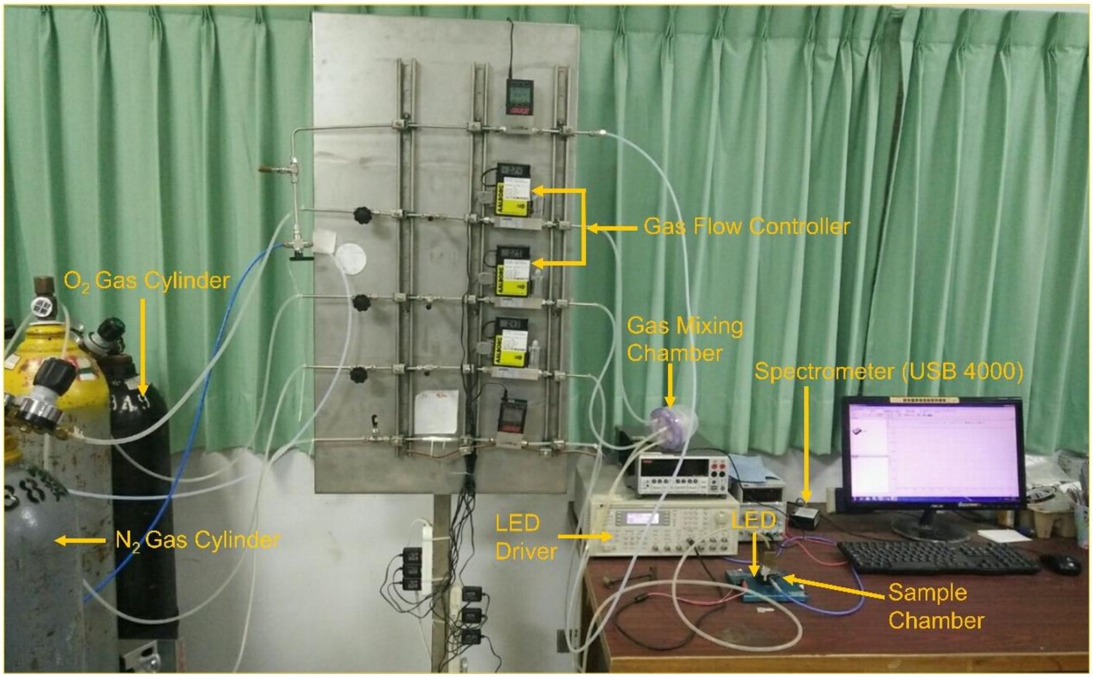

Figure Sa: Optical image of the experimental setup.

Estimation of surface area increment in AAO substrate:

The increase in surface area of the AAO substrate compared to the flat glass substrate is commensurate with the increase in fluorescence signal as explained here. Since, the AAO nanochannels are intrinsically arranged in hexagonal close- packed pattern, let's consider area of a hexagon as a unit area for the calculation. Assume, 'r' is the radius of the pores and 'l' is the effective length of the nanochannels contributing to the optical signal. Therefore, area of a hexagon without pores i.e. flat surface (denoted as A_f) is given by

$$A_f = \frac{3\sqrt{3}}{2}(4r)^2$$

and area of the hexagon with nanochannels (denoted as A_n) is given as

$$A_n = \frac{3\sqrt{3}}{2}(4r)^2 - 3\pi r^2 + 6\pi rl$$

Now, for r = 100 nm and l = 1 µm, the ratio of A_n / A_f is approximated to 5.3 satisfying the 5 times increase in fluorescence intensity. Note that, the indicator molecules are present throughout the entire nanochannels (~ 60 µm) which is much longer than 1 µm.

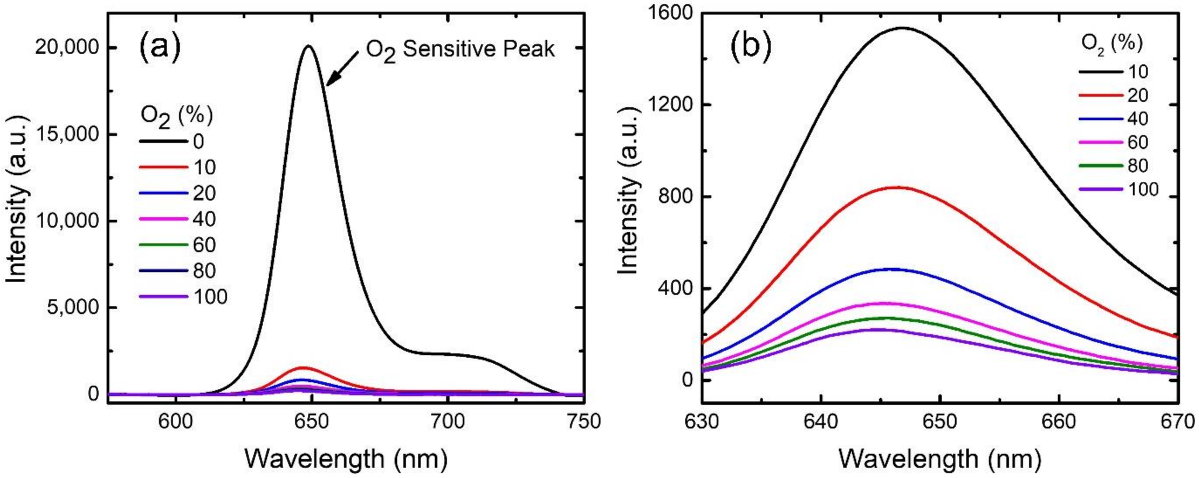

Figure S1. (a) Emission spectra of the PtTFPP molecule on glass substrate with different oxygen concentrations from 0-100%. (b) Enlarged spectra from Figure(a) for better visualization.

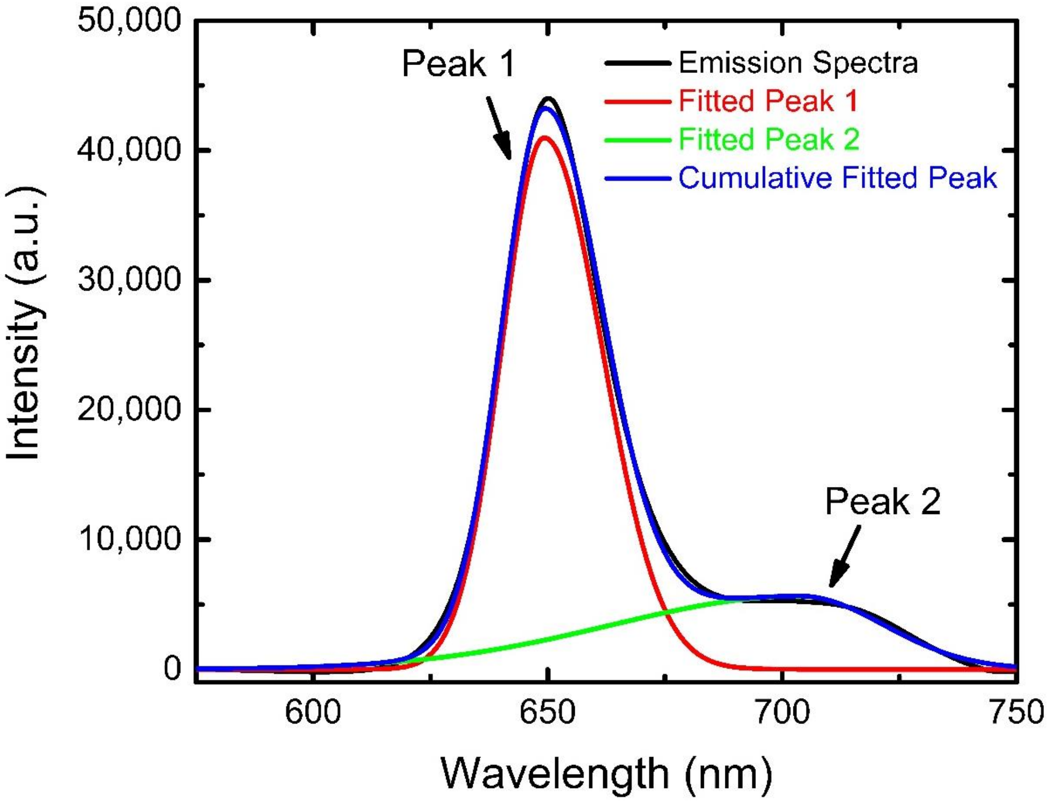

Figure S2. Gaussian fitting of a typical spectrum (PtTFPP molecules on an AAO membranes at 0% O_2) to extract actual peak intensities.

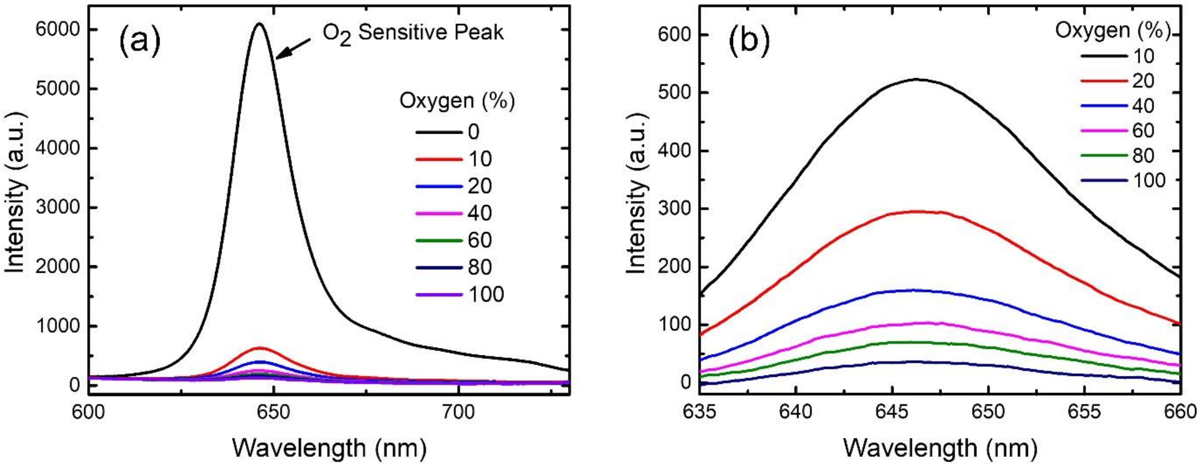

Figure S3. (a) Phosphorescent spectra of the PtOEP molecule on glass substrate with increasing steps of oxygen concentrations from 0-100%. (b) Enlarged spectra from Figure (a) for better visualization.

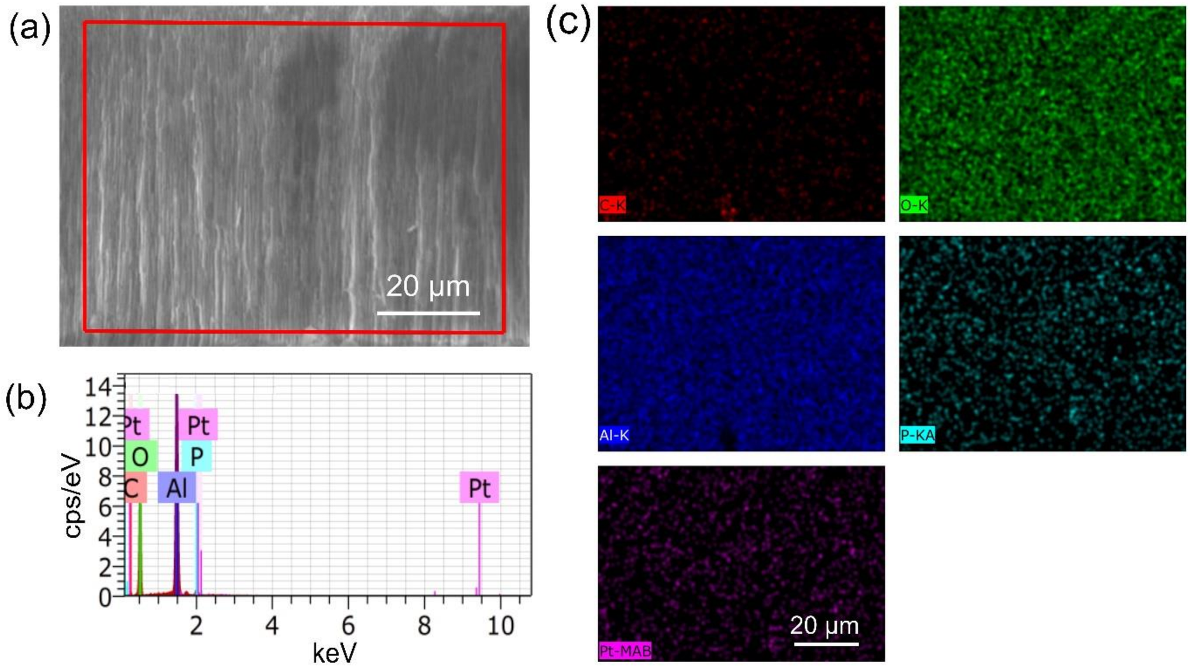

Figure S4. (a) Cross-sectional SEM image of the AAO nano-channels filled with PtOEP molecule. (b) EDS spectra revealing Al, O, C, Pt, and P peak separately. (c) EDS mapping on the area marked by the red square in Figure (a).

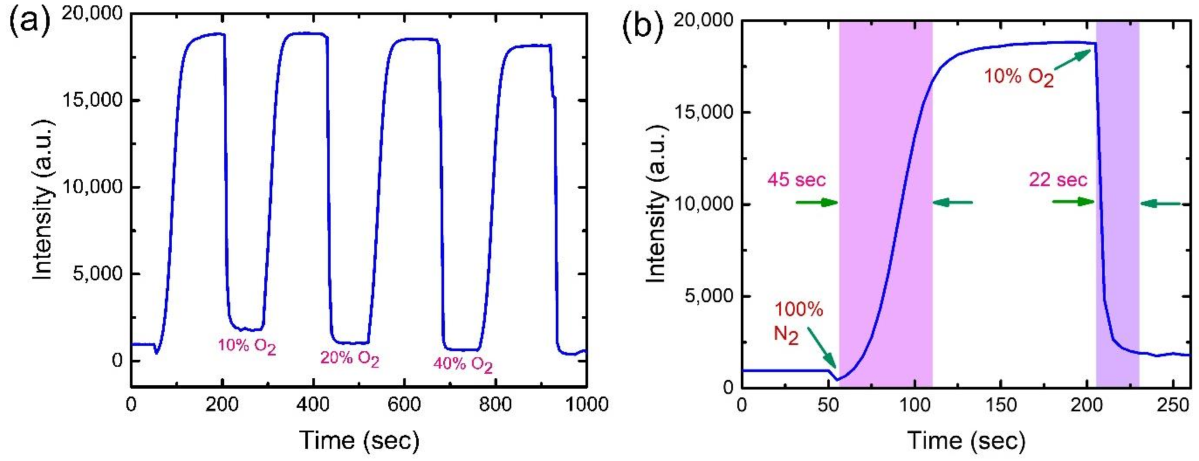

Figure S5. (a) Dynamic response of PtTFPP oxygen sensor on glass substrate with gradual change in O_2 concentrations. (b) Zoomed-in plot of Figure (a) to estimate the response and recovery time.

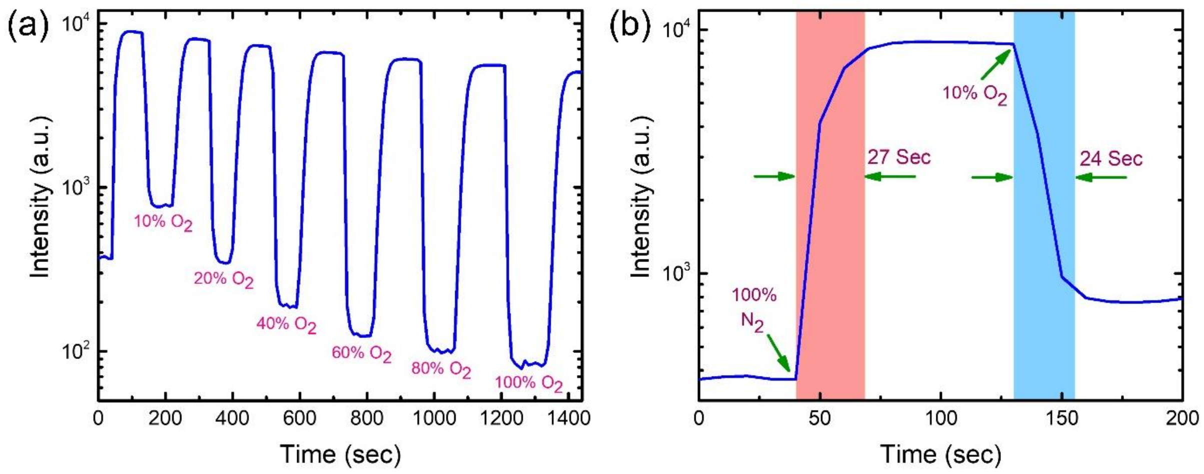

Figure S6. (a) Dynamic response of PtOEP oxygen sensor on glass substrate with systematically modified O_2 concentrations. (b) Zoomed-in plot of Figure (a) to estimate the response and recovery time.

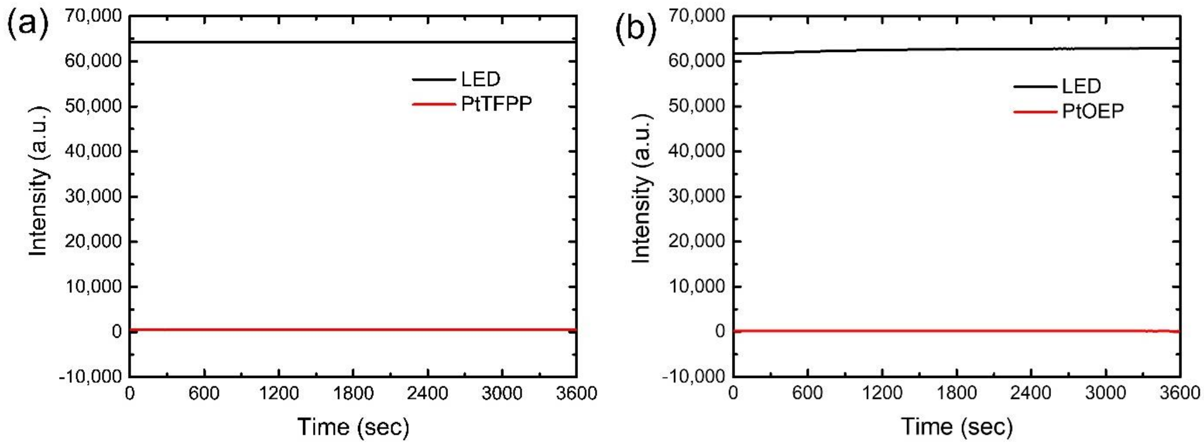

Figure S7. Photostability data of the oxygen sensors for (a) PtTFPP and (b) PtOEP on glass substrate under one-hour continuous illumination.

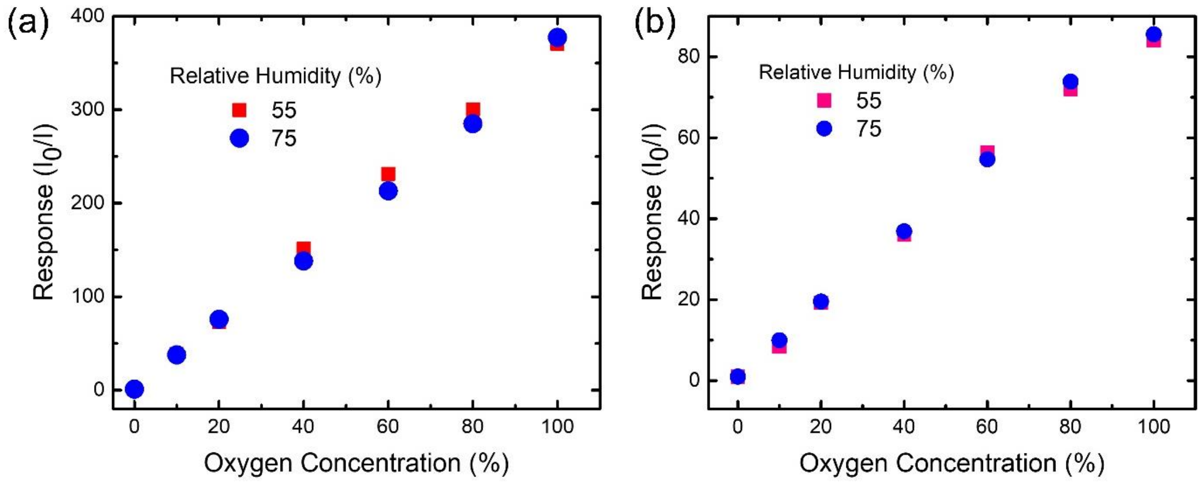

Figure S8. Response of the PtTFPP sensor fabricated on (a) AAO and (b) glass substrates at different relative humidity – low (RH 55%) and high (RH 75%).

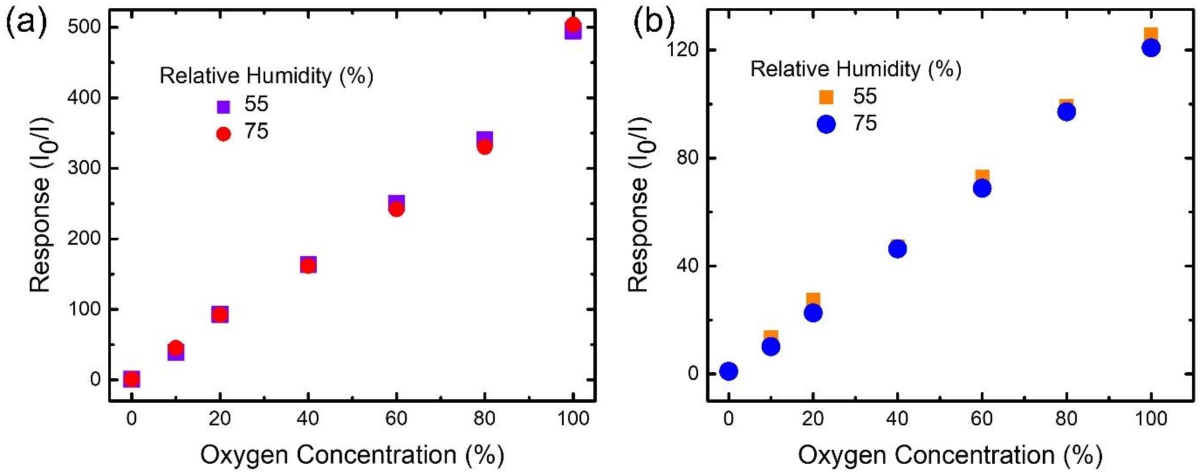

Figure S9. Response of the PtOEP sensor fabricated on (a) AAO and (b) glass substrates at different relative humidity – low (RH 55%) and high (RH 75%).

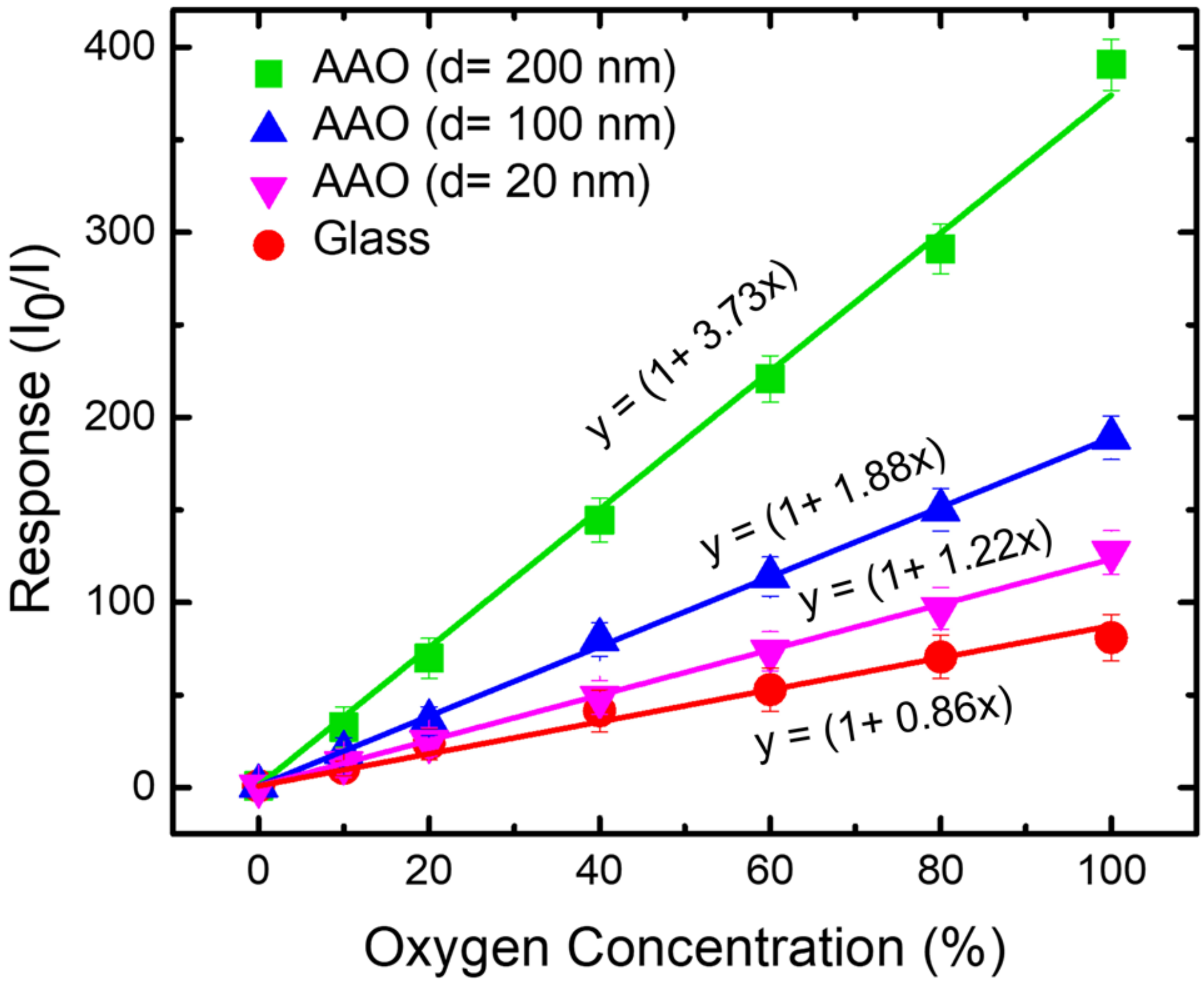

Figure S10. S-V plots comparing the responses (PtTFPP) from the AAO substrates with different pore diameters (200 nm, 100 nm, 20 nm) with that of the glass substrate.

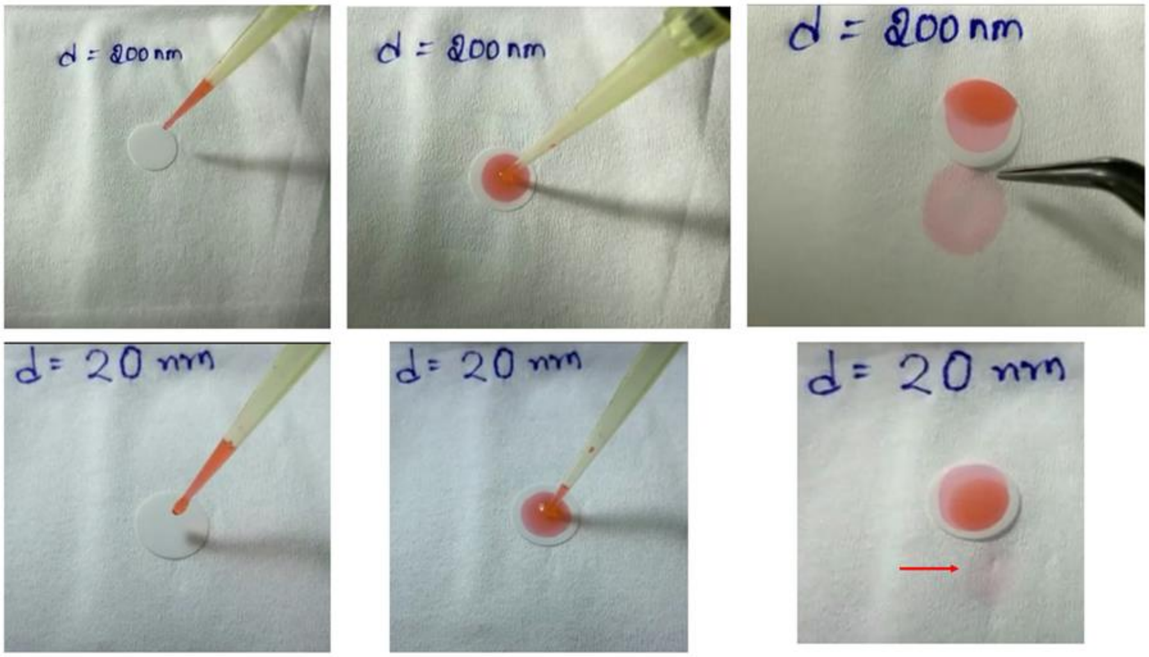

Figure S11. Snapshots demonstrating dye molecules oozing out of the nanochannels.
